# The Practice of Medicine; or a Treatise on Special Pathology and Therapeutics

**Published:** 1842-04

**Authors:** 


Art. VI.—*The Practice of Medicine; or, a Treatise on Special Pathology and Therapeutics.* By Robley Dunglison, M. D., Professor of the Institutes of Medicine, &c., in Jefferson Medical College, Philadelphia; Lecturer on Clinical Medicine, and attending Physician at the Philadelphia Hospital, &c., &c. In two volumes, pp. 572—750. Philadelphia: Lea & Blanchard, 1842.

Professor Dunglison is certainly a man of the most prodigious industry, to say nothing of his talents, which are doubtless of a high order. The present treatise constitutes the seventh that has been furnished by his prolific pen within the last twelve or thirteen years. His first production, entitled "Commentaries on Diseases of the Stomach and Bowels of Children," appeared in England in 1824, a short time before he emigrated from that country, of which he is a native, to the United States, to assume the duties of a teacher of medicine in the University of Virginia, to which he had been invited by President Jefferson, the distinguished founder of that excellent institution. Like the early efforts of Cooper and of Bulwer, the work in question fell still-born from the press; it was severely criticised by the reviewers, and no reprint of it has ever appeared in this country. All his other works, however, certainly the majority of them, have been well received by the profession, and one of them, "Human Physiology," has already reached the fourth edition. Scarcely forty-five years of age, he has published a greater number of works than any other physician on this side of the Atlantic, whether dead or living. To him writing seems to be no labor; books come to him almost at a call; he has only to say, "let there be a book, and a book there is." In addition to his various medical productions, Professor Dunglison has been editing for some time past a medical periodical, one of the most valuable, by the way, in the country, and has contributed some very learned articles to several of our literary reviews.

From the immense amount of labor, both intellectual and physical, which he has accomplished, one would suppose that the Professor was a thin, emaciated, Smike-like personage, or still worse, prematurely old and grey-headed, with a body bent like that of poor Scarron in the form of a Z. Not so, however. He is really a fine looking man, whose whole exterior is indicative of the most vigorous health, whose enbonpoint would do honor to an alderman, or even "my lord-mayor," who has none of that irritability which so much worries and frets the poet, especially those in high places—the garret and the attic, and whose social qualities are as rare as they are delightful. Notwithstanding his immense labor as an author, he possesses a sort of ubiquity truly remarkable. Go where you will in Philadelphia, you find the Professor. Does a stranger of distinction visit the city, he is the very first to call upon him; in the morning he is at the Blockley Hospital, or in attendance on his private patients; in the afternoon he saunters forth in pursuit of health or recreation; in the evening he is at a party, or perhaps at the rooms of the American Philosophical Society, of which he is the corresponding secretary, and one of the most useful and active members. But when, asks the reader, does the Professor find time to compose his works? Not certainly in the morning, nor in the afternoon, nor yet early in the evening; no, these hours are all occupied in other pursuits; it is late at night, at midnight, if you please, when other men are wrapt in sleep and dreaming, *not of what they have done, but of what they intend to do,* that he produces those works which have made his name so honorably known in America and Europe, and so closely identified it with the history of medical science and liteiature in the nineteenth century.

Having said thus much about the *man,* we need not make any particular comments on his recent *production,* as our object merely is to attract to it the attention of the profession. Like its predecessors, it bears the impress of sound judgment, profound research, and extensive acquaintance with the labors of others in the same department. Next to the "Physiology," it is decidedly the best and most finished of his works. The arrangement is admirable, and the whole is executed in a manner highly honorable to the talents and learning of the distinguished author. If we mistake not, it will be extensively sought after by the profession, and adopted by many of our schools as a text-book. That it will supersede most of the treatises on medicine now in use among us, there can be no doubt. The mechanical execution is worthy of the matter; the paper and typography are excellent; and the *tout ensemble* is such as to induce the conviction that, in book-making at least, we are but a step in arrear of old England herself.

